# Supplementary material for: Using the Maize Nested Association Mapping (NAM) Population to Partition Arbuscular Mycorrhizal Effects on Drought Stress Tolerance into Hormonal and Hydraulic Components
Source: Int J Mol Sci. 2022 Aug 29;23(17):9822. doi: 10.3390/ijms23179822 (PMC9456450; doi:10.3390/ijms23179822)
Supplement: Supplementary file 1 [file ijms-23-09822-s001.zip › Figure Supplementary S1 hormones.pdf]

**Figure S1.** Sap hormonal content (ABA, IAA, SA, JA and JA-Ile) in the whole maize NAM population cultivated under well-watered conditions or subjected to drought stress. Data represents the means of 6 values ± S.E.

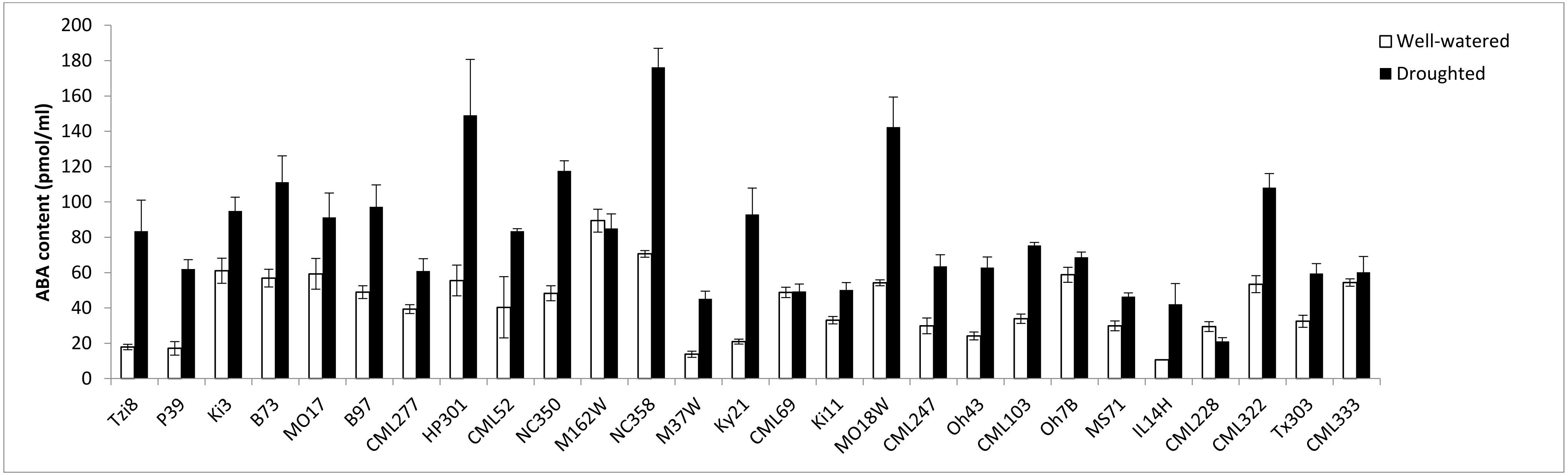


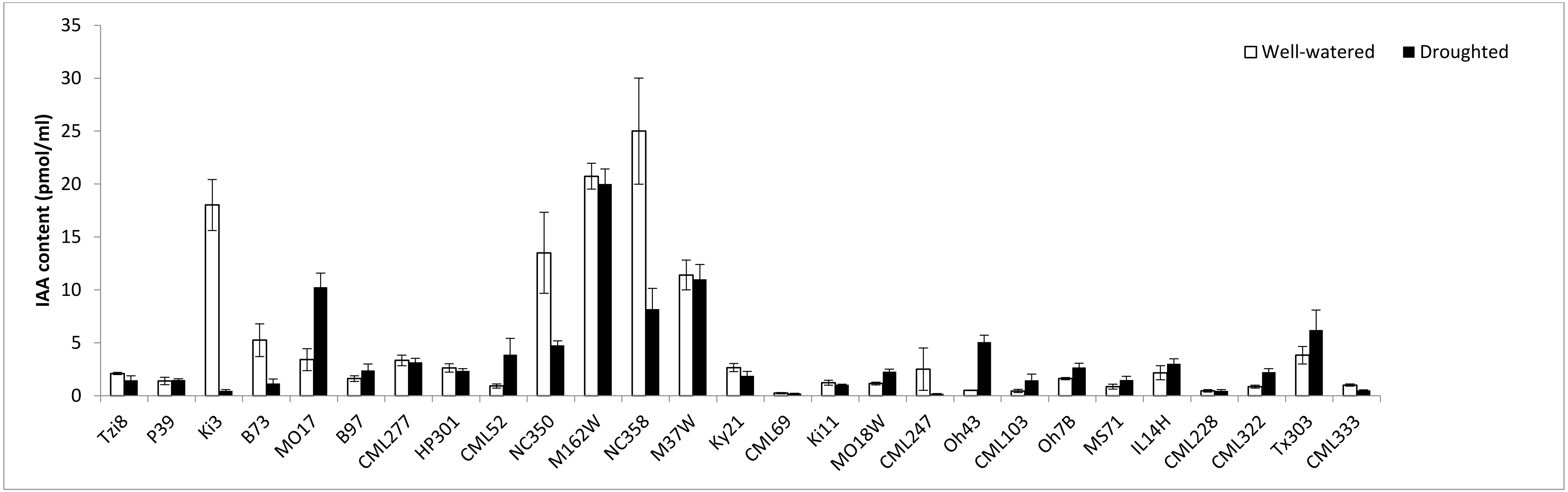

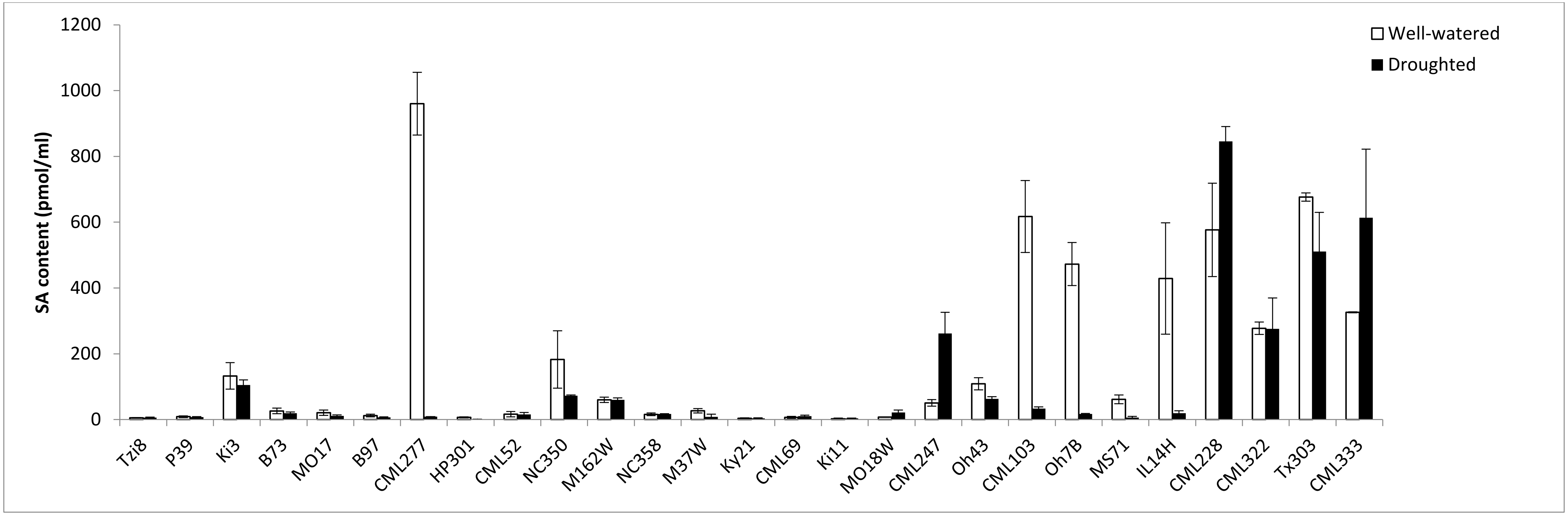

Well-watered
Droughted
SA content (pmol/ml)
0
200
400
600
800
1000
1200
Tzi8
P39
Ki3
B73
MO17
B97
CML277
HP301
CML52
NC350
M162W
NC358
M37W
Ky21
CML69
Ki11
MO18W
CML247
Oh43
CML103
Oh7B
MS71
IL14H
CML228
CML322
Tx303
CML333


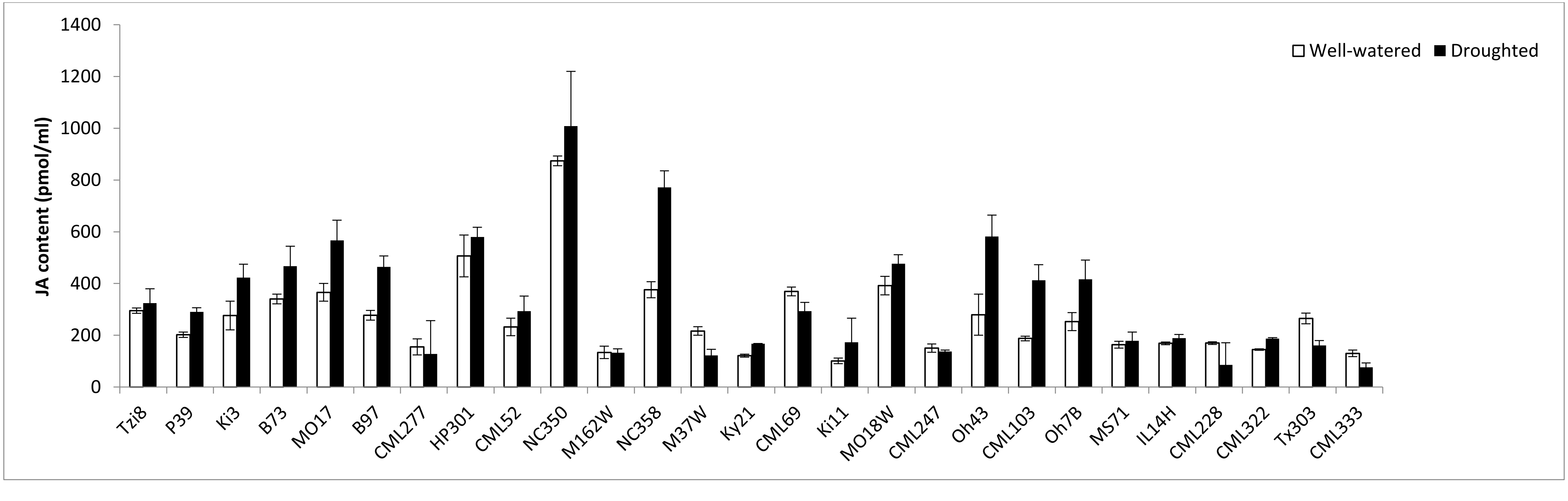

Well-watered
Droughted
JA content (pmol/ml)
0
200
400
600
800
1000
1200
1400
Tzi8
P39
Ki3
B73
MO17
B97
CML277
HP301
CML52
NC350
M162W
NC358
M37W
Ky21
CML69
Ki11
MO18W
CML247
Oh43
CML103
Oh7B
MS71
IL14H
CML228
CML322
Tx303
CML333

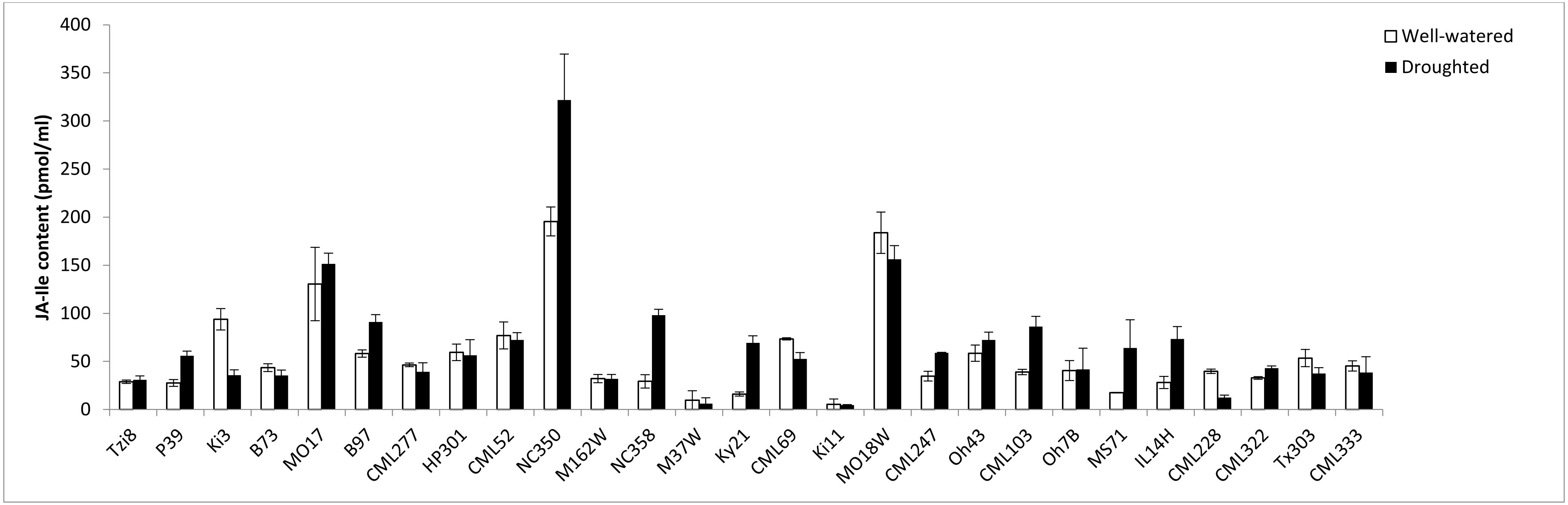
Well-watered
Droughted
JA-Ile content (pmol/ml)
400
350
300
250
200
150
100
50
0
Tzi8
P39
Ki3
B73
MO17
B97
CML277
HP301
CML52
NC350
M162W
NC358
M37W
Ky21
CML69
Ki11
MO18W
CML247
Oh43
CML103
Oh7B
MS71
IL14H
CML228
CML322
Tx303
CML333
