# Supplementary material for: Using the Maize Nested Association Mapping (NAM) Population to Partition Arbuscular Mycorrhizal Effects on Drought Stress Tolerance into Hormonal and Hydraulic Components
Source: Int J Mol Sci. 2022 Aug 29;23(17):9822. doi: 10.3390/ijms23179822 (PMC9456450; doi:10.3390/ijms23179822)
Supplement: Supplementary file 1 [file ijms-23-09822-s001.zip › Figure Supplementary S2 aquaporines.pdf]

Figure S2: Relative gene expression in eight maize aquaporin genes (*ZmPIP1;1, ZmPIP1;3, ZmPIP2;2, ZmPIP2;4, ZmTIP1;1, ZmTIP2;3, ZmTIP4;1 and ZmNIP2;1)* in six maize NAM lines (B73, Ky21, NC350, Tzi8, Ms71 and Mo18W) selected from experiment 1 and inoculated or not with an arbuscular mycorrhizal (AM) fungus. Plants were cultivated under well-watered conditions or subjected to drought stress. Data represents the means of 5 replicates ± S.E.

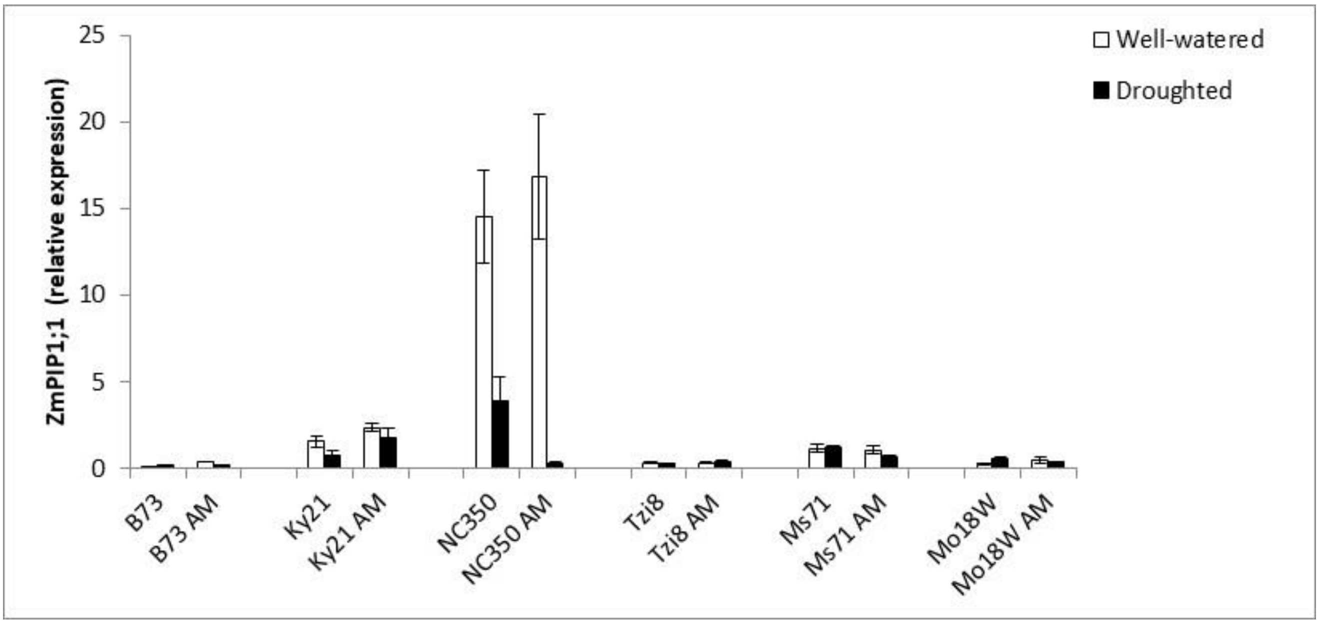

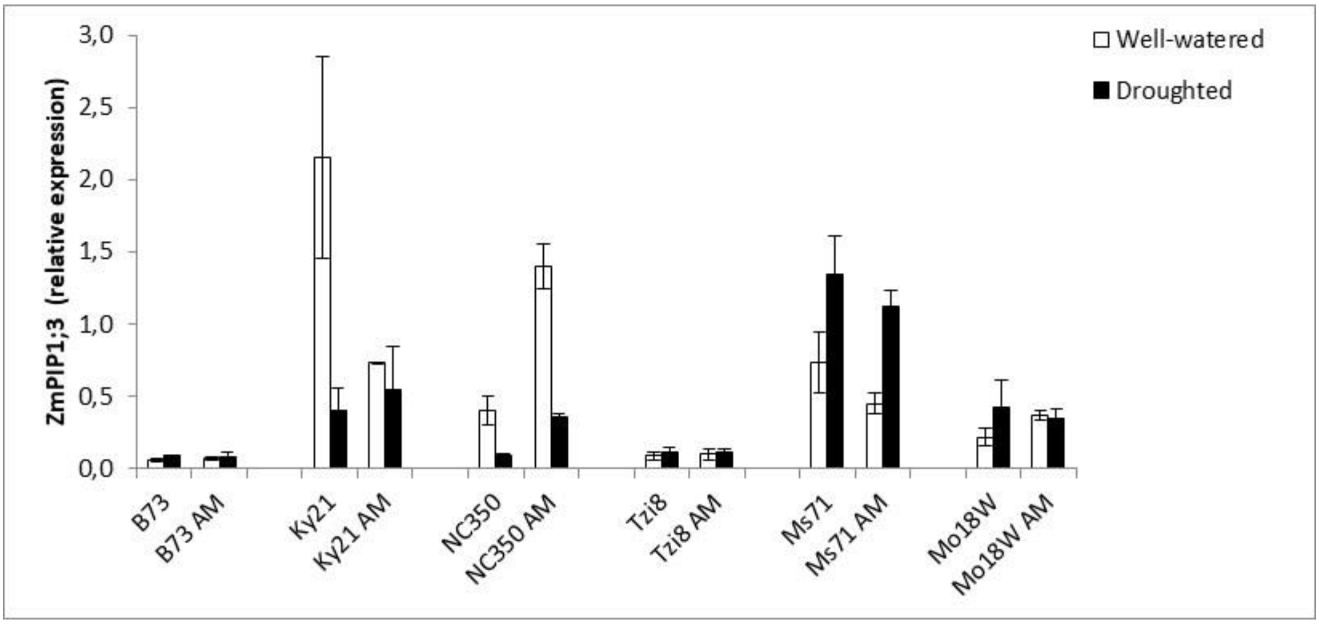

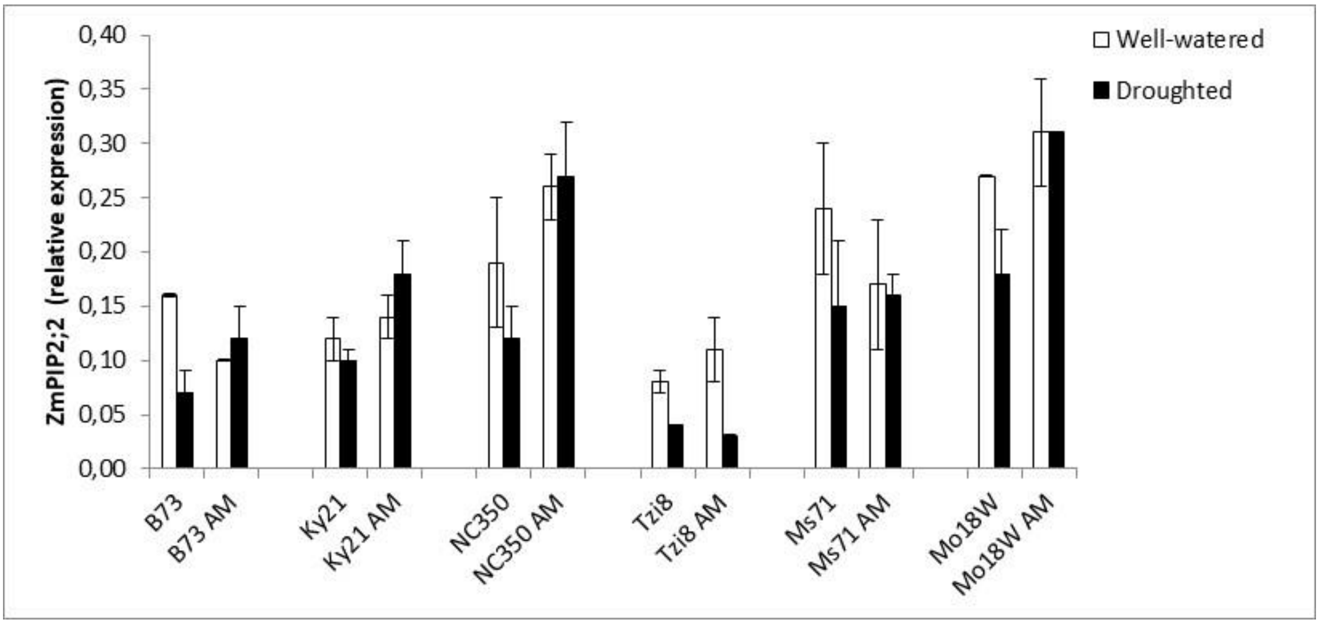

ZmPIP2;2 (relative expression)
0,40
0,35
0,30
0,25
0,20
0,15
0,10
0,05
0,00
Well-watered
Droughted
B73
B73 AM
Ky21
Ky21 AM
NC350
NC350 AM
Tzi8
Tzi8 AM
Ms71
Ms71 AM
Mo18W
Mo18W AM

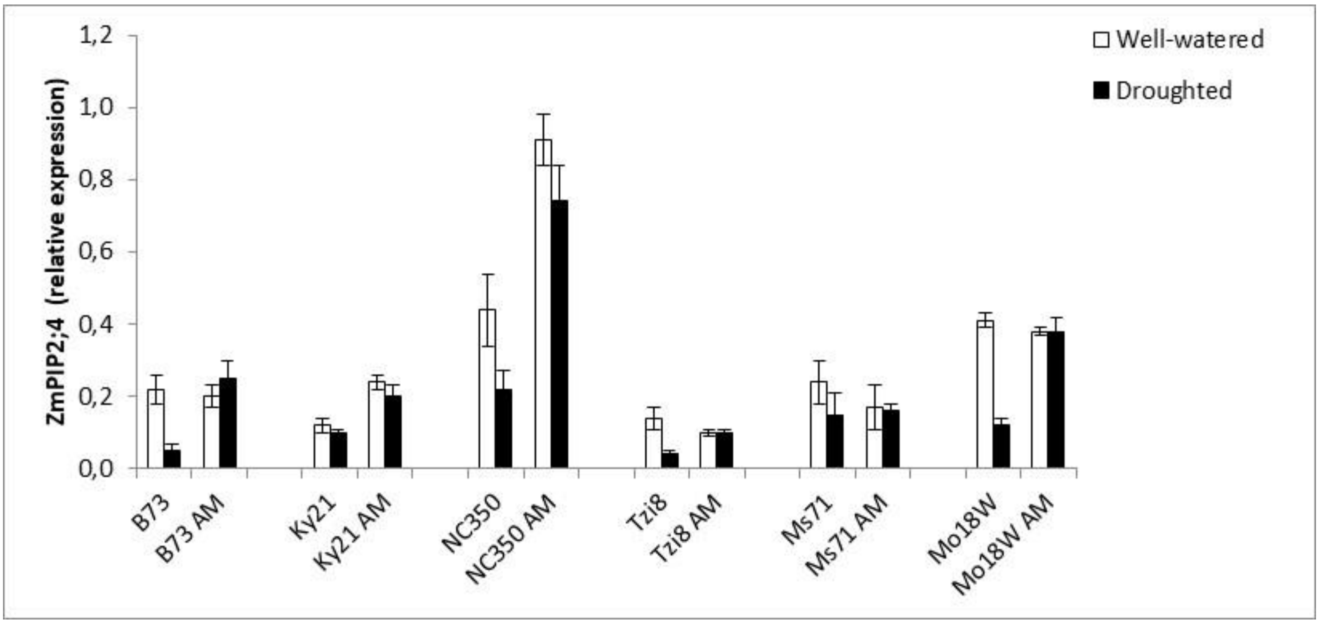

ZmPIP2;4 (relative expression)
1,2
1,0
0,8
0,6
0,4
0,2
0,0
Well-watered
Droughted
B73
B73 AM
Ky21
Ky21 AM
NC350
NC350 AM
Tzi8
Tzi8 AM
Ms71
Ms71 AM
Mo18W
Mo18W AM

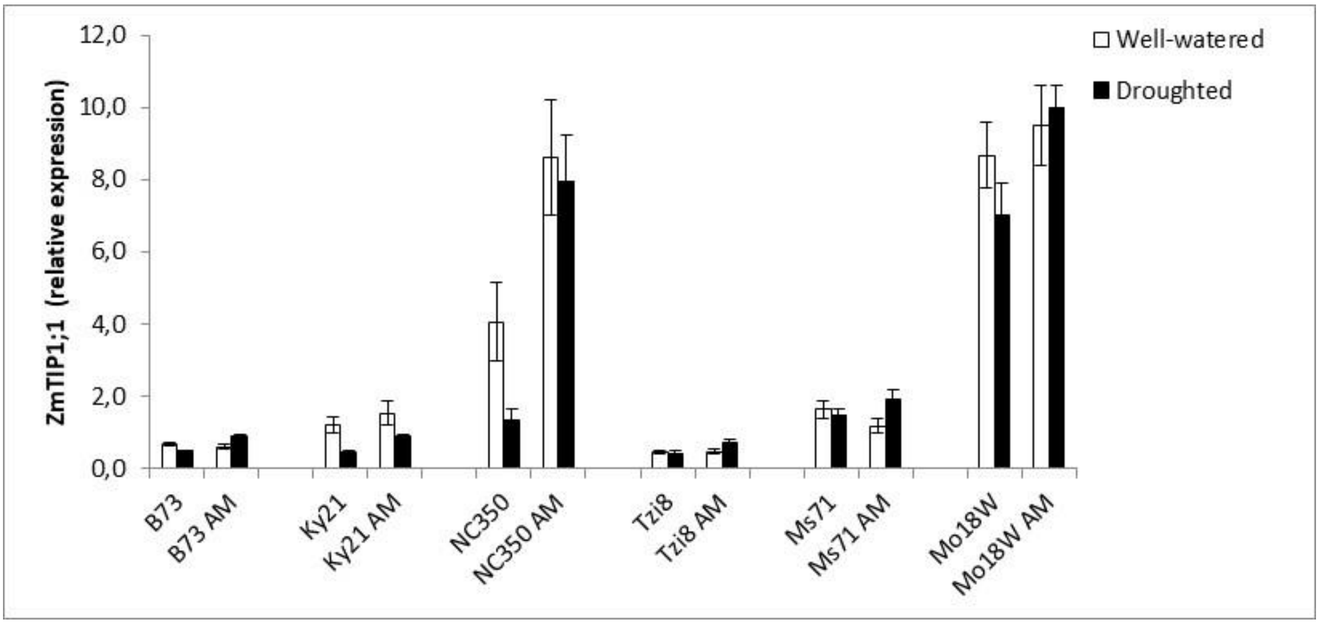

ZmTIP1;1 (relative expression)
12,0
10,0
8,0
6,0
4,0
2,0
0,0
Well-watered
Droughted
B73
B73 AM
Ky21
Ky21 AM
NC350
NC350 AM
Tzi8
Tzi8 AM
Ms71
Ms71 AM
Mo18W
Mo18W AM

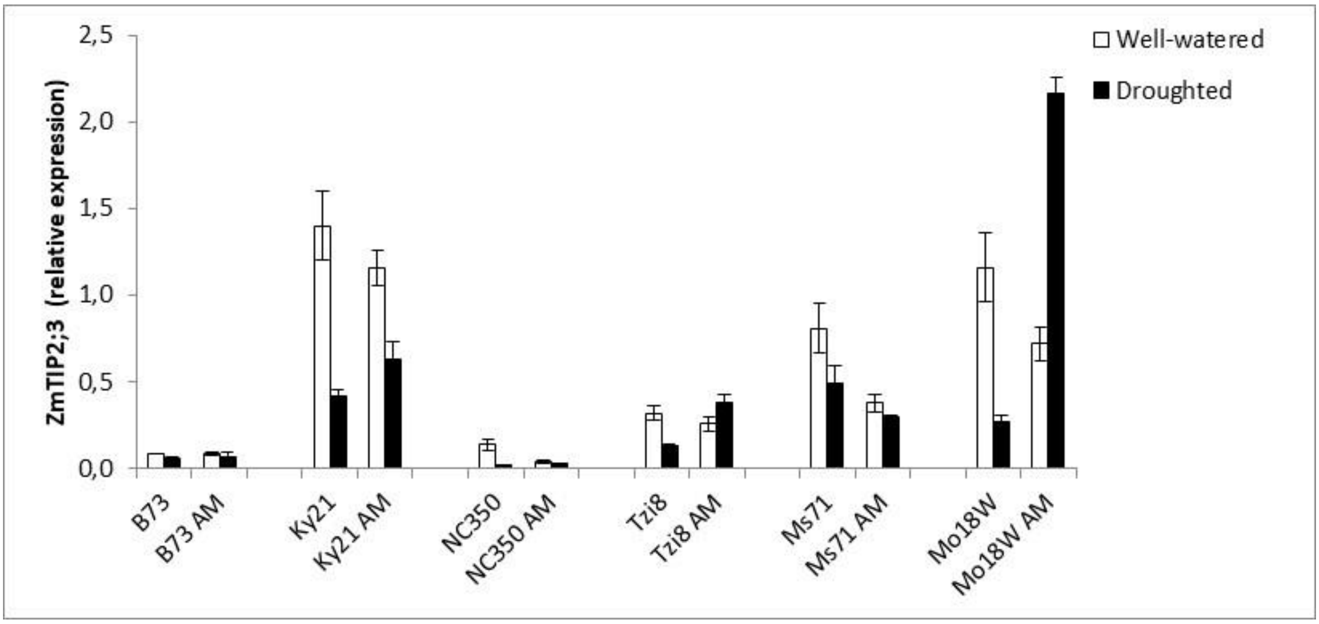
2,5
2,0
1,5
1,0
0,5
0,0
ZmTIP2;3 (relative expression)
Well-watered
Droughted
B73
B73 AM
Ky21
Ky21 AM
NC350
NC350 AM
Tzi8
Tzi8 AM
Ms71
Ms71 AM
Mo18W
Mo18W AM

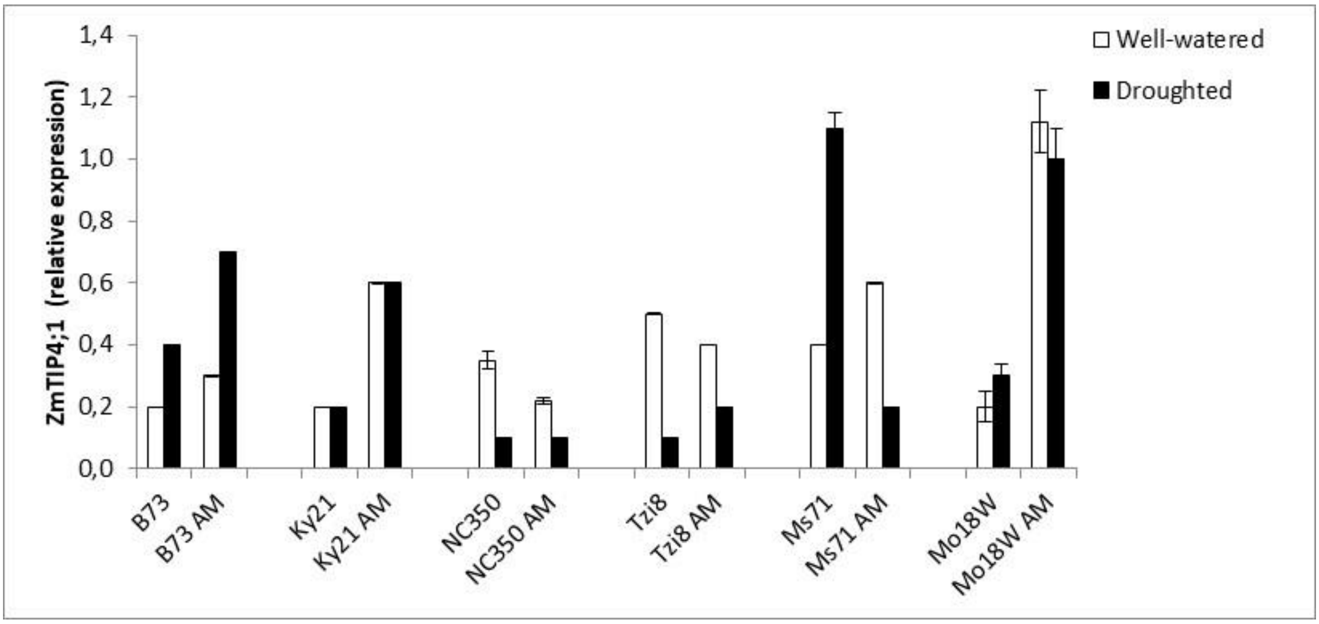
1,4
1,2
1,0
0,8
0,6
0,4
0,2
0,0
ZmTIP4;1 (relative expression)
Well-watered
Droughted
B73
B73 AM
Ky21
Ky21 AM
NC350
NC350 AM
Tzi8
Tzi8 AM
Ms71
Ms71 AM
Mo18W
Mo18W AM

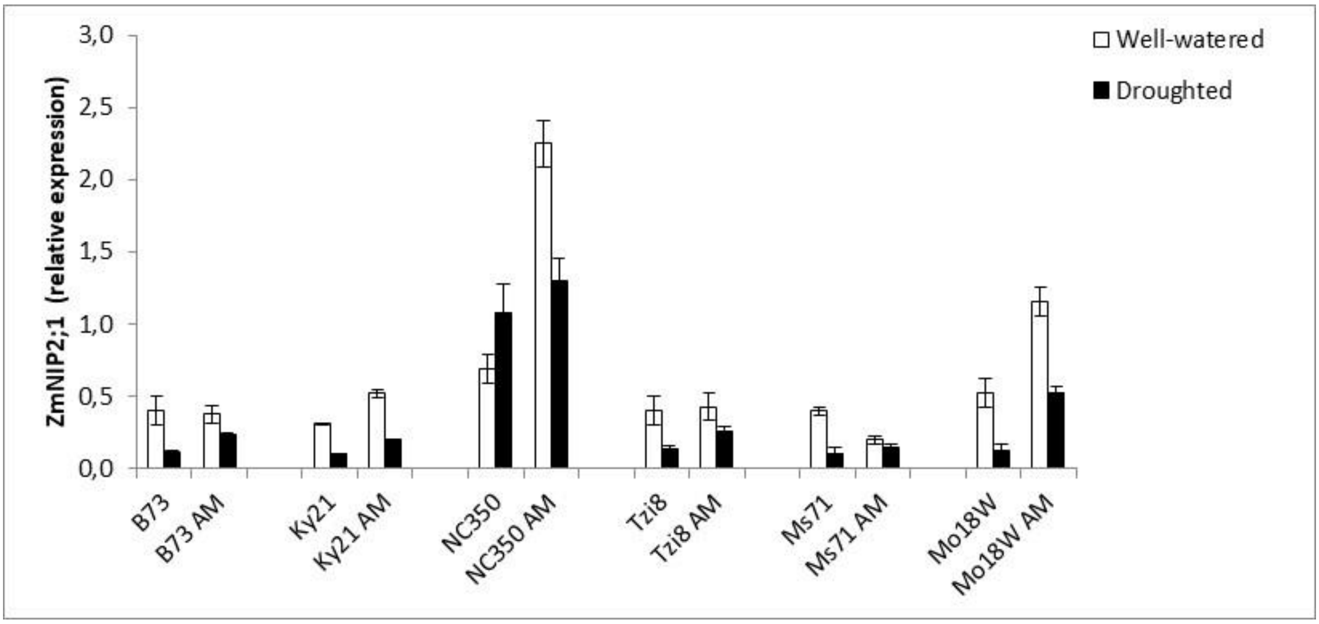
3,0
2,5
2,0
1,5
1,0
0,5
0,0
ZmNIP2;1 (relative expression)
Well-watered
Droughted
B73
B73 AM
Ky21
Ky21 AM
NC350
NC350 AM
Tzi8
Tzi8 AM
Ms71
Ms71 AM
Mo18W
Mo18W AM
