# Supplementary material for: Using the Maize Nested Association Mapping (NAM) Population to Partition Arbuscular Mycorrhizal Effects on Drought Stress Tolerance into Hormonal and Hydraulic Components
Source: Int J Mol Sci. 2022 Aug 29;23(17):9822. doi: 10.3390/ijms23179822 (PMC9456450; doi:10.3390/ijms23179822)
Supplement: Supplementary file 1 [file ijms-23-09822-s001.zip › Figure Supplementary S3 hormones.pdf]

Figure S3. Sap hormonal content (ABA, IAA, SA, JA and JA-Ile) in six maize NAM lines (B73, Ky21, NC350, Tzi8, Ms71 and Mo18W) selected from experiment 1 and inoculated or not with an arbuscular mycorrhizal (AM) fungus. Plants were cultivated under well-watered conditions or subjected to drought stress. Data represents the means of 6 values ± S.E.

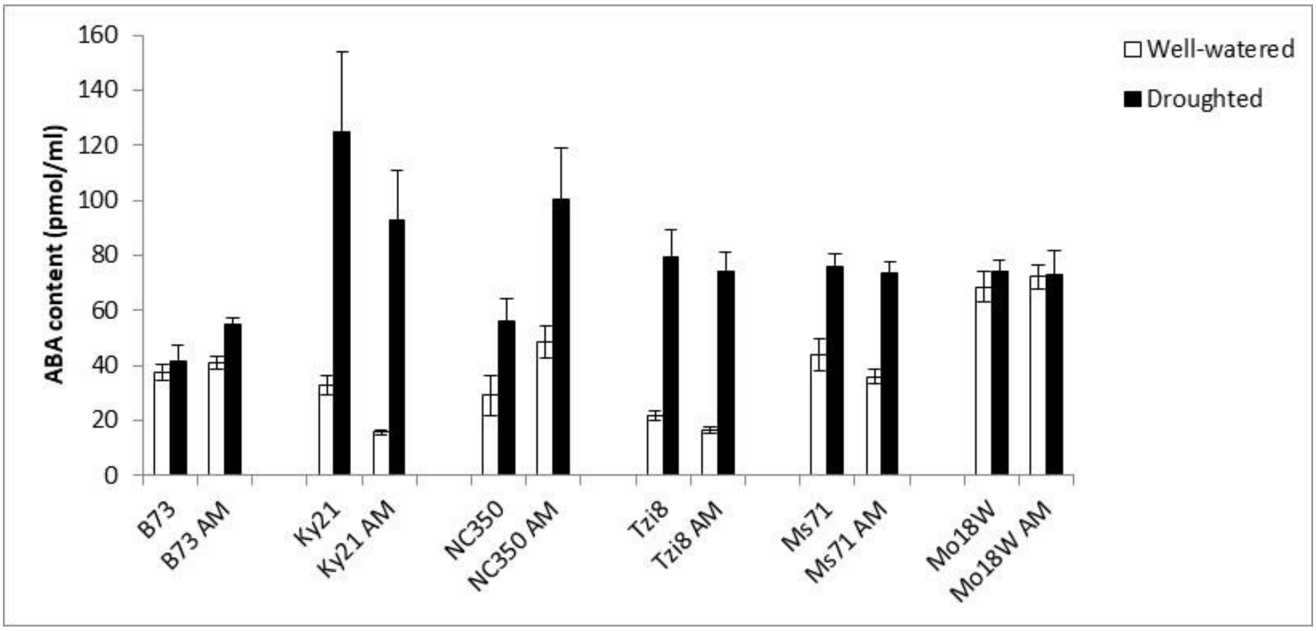

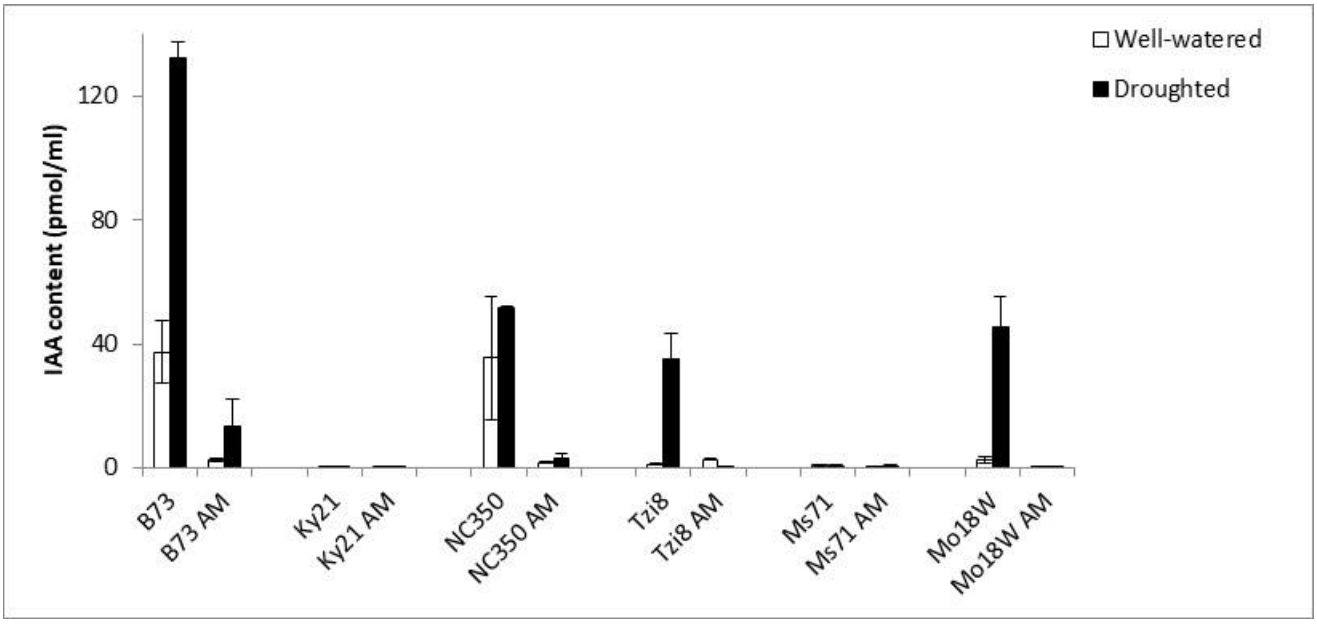

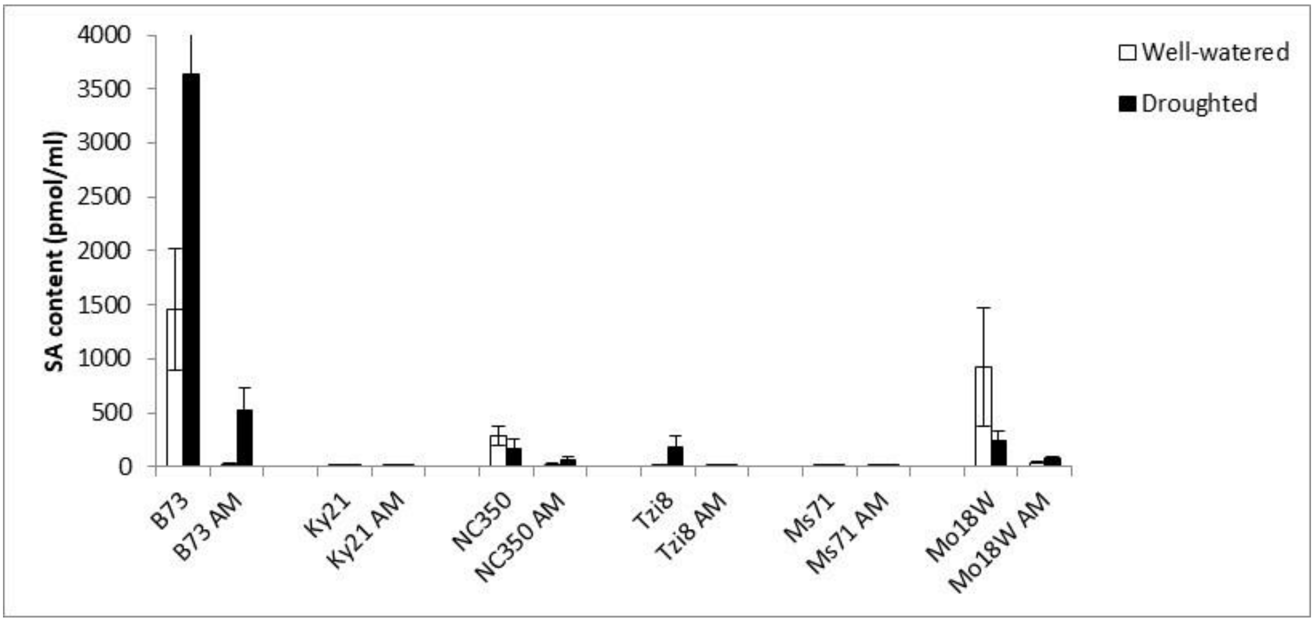
SA content (pmol/ml)
4000
3500
3000
2500
2000
1500
1000
500
0
Well-watered
Droughted
B73
B73 AM
Ky21
Ky21 AM
NC350
NC350 AM
Tzi8
Tzi8 AM
Ms71
Ms71 AM
Mo18W
Mo18W AM

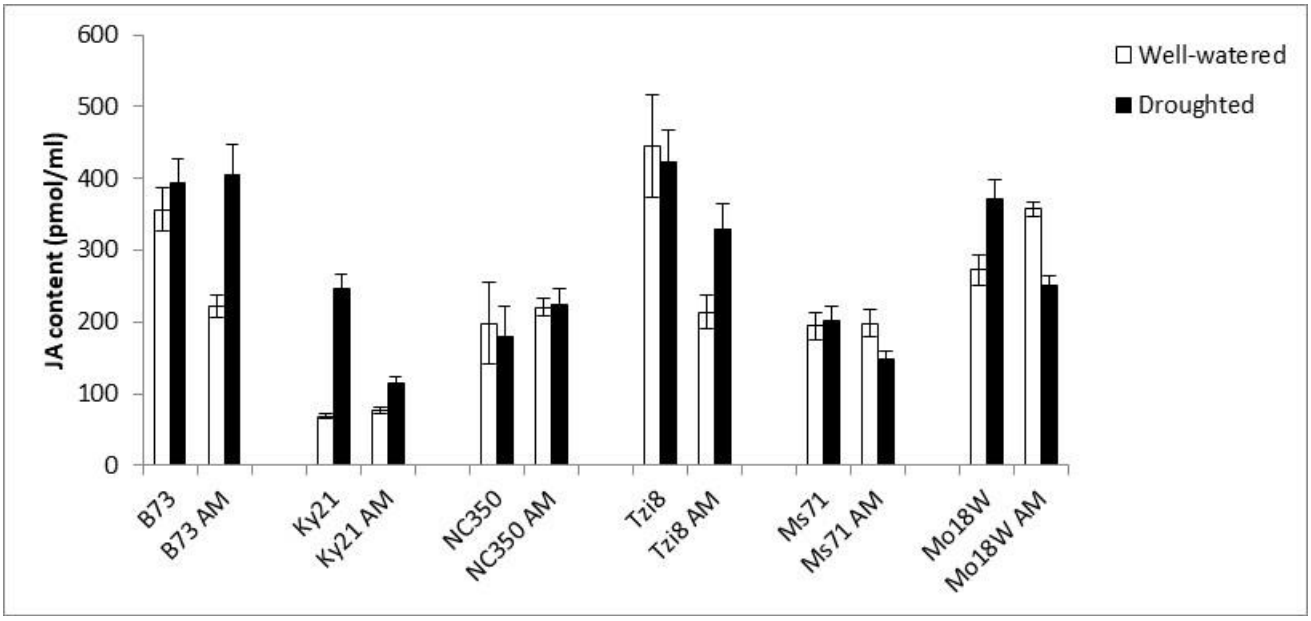
JA content (pmol/ml)
600
500
400
300
200
100
0
Well-watered
Droughted
B73
B73 AM
Ky21
Ky21 AM
NC350
NC350 AM
Tzi8
Tzi8 AM
Ms71
Ms71 AM
Mo18W
Mo18W AM

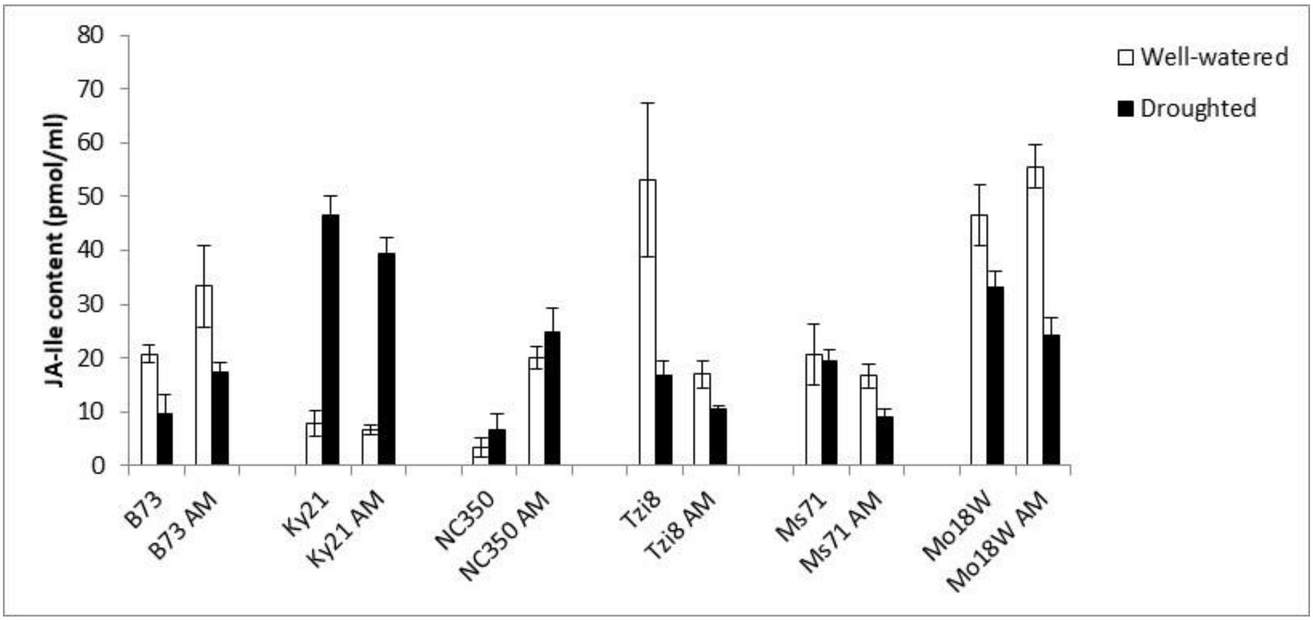
JA-Ile content (pmol/ml)
80
70
60
50
40
30
20
10
0
Well-watered
Droughted
B73
B73 AM
Ky21
Ky21 AM
NC350
NC350 AM
Tzi8
Tzi8 AM
Ms71
Ms71 AM
Mo18W
Mo18W AM
