# Supplementary material for: Using the Maize Nested Association Mapping (NAM) Population to Partition Arbuscular Mycorrhizal Effects on Drought Stress Tolerance into Hormonal and Hydraulic Components
Source: Int J Mol Sci. 2022 Aug 29;23(17):9822. doi: 10.3390/ijms23179822 (PMC9456450; doi:10.3390/ijms23179822)

Figure S4: Percentage of mycorrhizal root length in six maize NAM lines (B73, Ky21, NC350, Tzi8, Ms71 and Mo18W) selected from experiment 1 and inoculated with an arbuscular mycorrhizal (AM) fungus. Plants were cultivated under well-watered conditions or subjected to drought stress. Data represents the means of 5 replicates ± S.E.

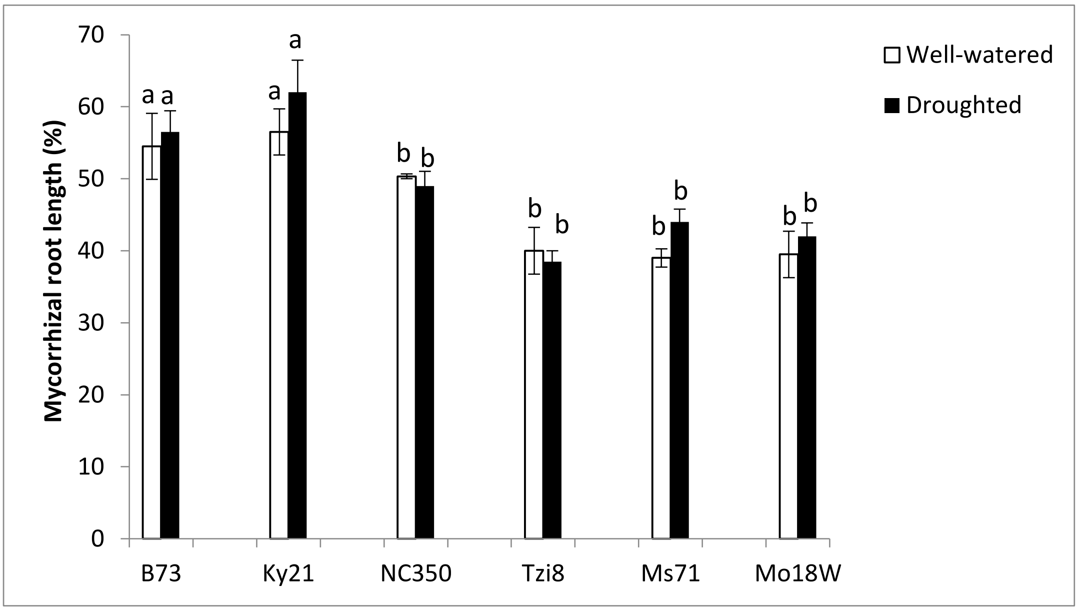

Supplement: Supplementary file 1 [file ijms-23-09822-s001.zip › Figure Supplementary S4 AM colonization.pdf]
